# Preservation effect of imeglimin on pancreatic β-cell mass: Noninvasive evaluation using ^111^In-exendin-4 SPECT/CT imaging and the perspective of mitochondrial involvements

**DOI:** 10.3389/fendo.2022.1010825

**Published:** 2022-09-29

**Authors:** Muhammad Fauzi, Takaaki Murakami, Hiroyuki Fujimoto, Ainur Botagarova, Kentaro Sakaki, Sakura Kiyobayashi, Masahito Ogura, Nobuya Inagaki

**Affiliations:** ^1^ Department of Diabetes, Endocrinology, and Nutrition, Graduate School of Medicine, Kyoto University, Kyoto, Japan; ^2^ Radioisotope Research Center, Agency of Health, Safety, and Environment, Kyoto University, Kyoto, Japan

**Keywords:** imeglimin, beta cell mass, SPECT/CT, BCM preservation, mitochondria

## Abstract

Progressive loss of β-cell mass (BCM) has a pernicious influence on type 2 diabetes mellitus (T2DM); evaluation of BCM has conventionally required an invasive method that provides only cross-sectional data. However, a noninvasive approach to longitudinal assessment of BCM in living subjects using an indium 111–labeled exendin-4 derivative ([Lys12(^111^In-BnDTPA-Ahx)]exendin-4) (^111^In-exendin-4) has been developed recently. Imeglimin is a novel antidiabetic agent that is reported to improve glycemic control and glucose-stimulated insulin secretion (GSIS) *via* augmentation of mitochondrial function. However, the influence of imeglimin on BCM is not fully understood. We have investigated the effects of imeglimin on BCM *in vivo* in prediabetic db/db mice using a noninvasive ^111^In-exendin-4 single-photon emission computed tomography/computed tomography (SPECT/CT) technique. During the 5-week study period, imeglimin treatment attenuated the progression of glucose intolerance, and imeglimin-treated mice retained greater BCM than control, which was consistent with the results of ^111^In-exendin-4 SPECT/CT scans. Furthermore, immunohistochemical analysis revealed reduced β-cell apoptosis in the imeglimin-treated db/db mice, and also lowered release of cytosolic cytochrome c protein in the β cells. Furthermore, electron microscopy observation and membrane potential measurement revealed improved structural integrity and membrane potential of the mitochondria of imeglimin-treated islets, respectively. These results demonstrate attenuation of progression of BCM loss in prediabetic db/db mice partly *via* inhibition of mitochondria-mediated apoptosis.

## 1 Introduction

One of the major pathogenic features of type 2 diabetes mellitus (T2DM) is progressive loss of β-cell mass (BCM) (1, 2). Preserving BCM is therefore a promising strategy for long-term management of T2DM as well as its prevention (3). Understanding BCM loss might facilitate diagnosis and treatment of T2DM as well as clarify its pathophysiology in the disease (4, 5). However, the longitudinal changes of BCM in patients with T2DM have not fully investigated due to the lack of a practical noninvasive technique for assessing BCM (4). To overcome the limitations of the conventional histological method of BCM analysis, which is invasive and can provide only cross-sectional data (5, 6), the strategy to evaluate BCM by β cell-specific imaging using nuclear medicine techniques has been proposed (4, 5). Especially, exendin-based glucagon-like peptide-1 receptor (GLP-1R)-targeted imaging has emerged as a promising tool of noninvasive evaluation of BCM (5). A method employing an indium 111–labeled exendin-4 derivative ([Lys12(^111^In-BnDTPA-Ahx)]exendin-4) (^111^In-exendin-4) has been developed (5, 7). Its emission can be traced and measured using a single photon emission computed tomography (SPECT) scan (7, 8). Because it is non-invasive and repeatable, longitudinal changes of BCM can be evaluated in a living subject (4, 8–10).

In glucose homeostasis, the mitochondrion plays an essential role as critical cellular pathways such as energy substrate metabolism, reactive oxygen species (ROS) production, and apoptosis in various tissues including pancreatic β cells (11, 12). As observed in mitochondrial diabetes mellitus, mitochondrial dysfunction can be responsible for the impairment of glucose-stimulated insulin secretion (GSIS) and insulin sensitivity in T2DM (11–14). Therefore, mitochondria should be a promising therapeutic target of T2DM (11, 12). Imeglimin is a novel oral antidiabetic agent that is first of a new class called *glimins* (15). Imeglimin exerts its beneficial effects on glucose metabolism in T2DM by potentially modulating the mitochondrial functions of multiple tissues, including pancreas, liver and muscle (16, 17). It has been reported that improves NAD^+^/NADH ratio *via* salvage pathway and modulates Ca^2+^ release from endoplasmic reticulum (ER). Imeglimin is also known to optimize mitochondrial respiration, thus reduce ROS and increase ATP production (17). In pancreatic β cells, the mechanism of action involves amplification GSIS (18) and amelioration of β-cell apoptosis (19). Although previous findings suggest that imeglimin has beneficial effects on BCM, its effect on longitudinal changes of BCM and an underlying mechanism have not been investigated. In the present study, we investigated the longitudinal effect of imeglimin on BCM *in vivo* in prediabetic db/db mice by using noninvasive ^111^In-exendin-4 SPECT/CT imaging.

## 2 Materials and methods

### 2.1 Animals

Male C57BLKS/J Iar-+Lepr^db^/+Lepr^db^ mice (db/db) were purchased from Charles River (Tokyo, Japan). The Lepr^db/db^ (db/db) mouse is a diabetic model with obesity (20), in which rapidly decreased BCM (21) is accompanied in pancreatic β cells by a diminished number of mitochondrial organelles (22). Male animals were housed in a temperature-maintained chamber under 14/10 light/dark cycles with free access to food and water unless stated otherwise. The animal study was approved by The Committee of Animal Care and Use, Kyoto University (approval No. MedKyo 21508, 22222, 22227).

### 2.2 *In vivo* longitudinal evaluation of BCM

#### 2.2.1 Study design

**Figure 1:**
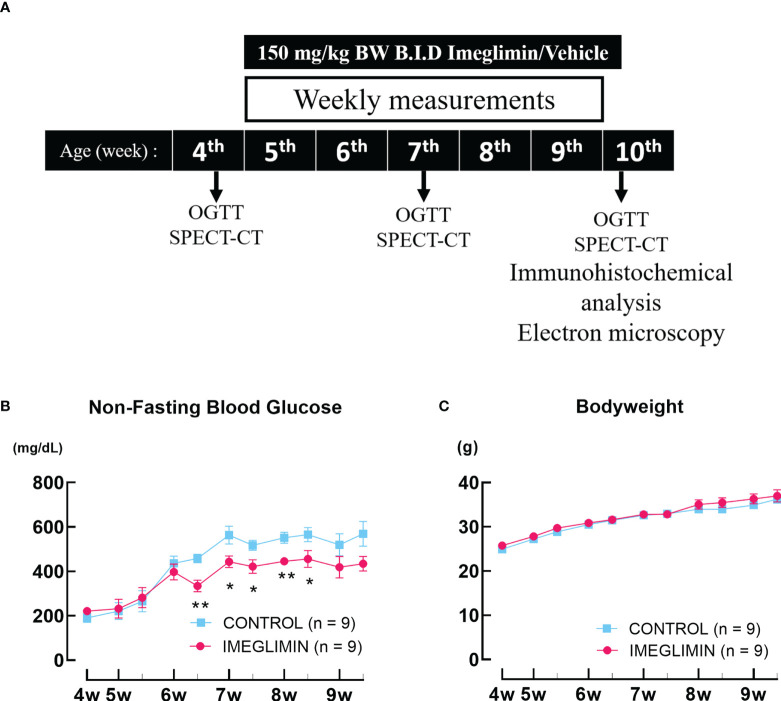
**(A)** Study design. **(B)** Non-fasting blood glucose and **(C)** body weight recorded twice a week during the observation period. The db/db mice are represented as follows: control, cyan squares (n = 9); imeglimin-treated, magenta circles (n = 9). Data are presented as mean ± SEM. Control vs imeglimin-treated, *p < 0.05, **p < 0.01.

Db/db mice at 4 weeks of age were divided into 2 groups of 9 mice: imeglimin-treated and control (vehicle-treated) not having a significant difference in body weight or blood glucose level. Interventions were started at the age of 5 weeks since db/db mice showed robust loss of BCM even in the very early phase of diabetes onset (4, 10) and continued for 5 weeks. The mice were orally administered 150 mg/kg body weight of imeglimin (Sumitomo pharma Co., Ltd., Osaka, Japan) or vehicle [0.5% methylcellulose (catalog no. 133-17815; Fujifilm, Osaka, Japan)] twice a day (B.I.D) for 5 weeks. During the study period, non-fasting blood glucose level and body weight were recorded. The glucose oxidase method (GT-1670; Arkray, Kyoto, Japan) was employed to determine the non-fasting blood glucose level. Oral glucose tolerance tests (OGTTs) were conducted at 4 (baseline), 7, and 10 weeks of age. ^111^In-exendin-4 SPECT/CT scans were performed to evaluate BCM at the same intervals on 3 mice for each group. Following the completion of the SPECT/CT scans, immunohistochemical analyses of BCM as well as proliferation and apoptosis of β cells were performed on 6-7 mice for each group. Transmission electron microscopy (TEM) was used to characterize the structure of the mitochondria in at least 10 pancreatic β cells collected from the mice of each group. The mouse used for each evaluation was initially allocated at the beginning of the observation period and was not altered during the study. The study design was represented in **Figure 1**.

#### 2.2.2 Oral glucose tolerance test

Oral glucose tolerance tests (OGTTs) were performed after16-hour fast and 24-hour agent-washout period. Glucose was administered orally by gavage (2 g glucose/kg body weight). Blood was collected *via* the tail vein at 0, 15, 30, 60, and 120 minutes. Blood glucose level was measured as mentioned; plasma insulin levels were measured using an Ultra Sensitive Mouse Insulin ELISA kit (catalog no. M1104; RRID: AB_2811268; Morinaga Institute of Biological Science, Yokohama, Japan).

#### 2.2.3 *In vivo* ^111^In-exendin-4 SPECT/CT

^111^In-exendin-4 SPECT/CT was performed on both imeglimin-treated and control mice at 4, 7, and 10 weeks of age. The ^111^In-exendin-4 probe [Lys12(111In-BnDTPA-Ahx)]exendin-4 was synthesized by attaching isothiocyanate-benzyl-DTPA (BnDTPA) and 6-aminohexanoic acid (Ahx) to an ϵ-amino group at the lysine-12 residue on exendin-4 (7). Approximately 3.0 MBq/mouse of ^111^In-exendin-4 was administered *via* tail vein 30 min before the SPECT/CT scan, which was conducted using Triumph LabPET12/SPECT4/CT (TriFoil Imaging Inc., Chatsworth, CA) (8). Pancreatic uptake was analyzed using Amira software, version 5.6.0 (FEI Visualization Sciences Group, Düsseldorf, Germany) from the SPECT/CT images as previously reported (8, 9). The region of interest was selected from the area between lungs and bladder. To avoid renal influence due to probe excretion, renal ROI determined from the CT image plus 2.7 mm surrounding the kidney were excluded. Then, the voxels with remaining signals in the abdominal space were counted as pancreatic uptake through the comparison of the CT image (8). The pancreatic uptake values (%ID/g) were calculated by normalizing the percentage of pancreatic radioactivity (Bq/g) in response to the injected probe (Bq) with the weight of the pancreatic tissue (g) (4, 8).

#### 2.2.4 Immunohistochemical analysis of BCM with β-cell proliferation and apoptosis

Immediately after the last of the SPECT/CT procedures, the mouse was sacrificed and the pancreas was resected. The organ was weighted, spread on mesh, and fixed in 4% paraformaldehyde solution at 4°C. For BCM histological analysis, 10 sets of formalin-fixed paraffin-embedded sections (4 μm per section; 100 μm between each set) were stained with anti-insulin primary antibody (1:100; catalog no. sc-9168; RRID: AB_2126540; Santa Cruz Biotechnology, Santa Cruz, CA) and Alexa Fluor 488 secondary antibody (1:200; catalog no. A11008; RRID: AB_143165; Thermo Fisher Scientific, Waltham, MA), as previously reported (4, 8, 9). For each set, a slide was stained with hematoxylin and eosin to calculate the whole section area. The stained slides were analyzed using a fluorescence microscope (BZ-X700; Keyence, Osaka, Japan). BCM was calculated by dividing the insulin-positive area by the whole section area and multiplying by the pancreas weight (mg) (4).

To assess cell proliferation, slides were stained with anti-insulin antibody (1:100; catalog no. ab7842; RRID: AB_306130; Abcam, Cambridge, MA) and anti-Ki67 antibody (1:100; catalog no. ab15580; RRID: AB_443209; Abcam) followed by staining with Alexa Fluor 488 antibody (1:200; catalog no. A11073; RRID: AB_2534117; Abcam) and Alexa Fluor 546 antibody (1:200; catalog no. A11035; RRID: AB_2534093; Thermo Fisher Scientific). The nuclei were stained with 4’,6-Diamidino-2-phenylindole (DAPI). The slides were mounted with VECTASHIELD (catalog no. H1000; RRID: AB_2336789; Vector Laboratory, Newark, CA). The proliferation rate was calculated as the ratio of insulin/Ki67/DAPI co-positive cells to total insulin-positive cells (23). To examine the apoptosis rate, the slides were stained using DeadEnd™ Fluorometric TUNEL System (catalog no. G3250, Promega, Madison, WI), according to the manufacturer’s manual, as well as anti-insulin antibody and DAPI with Alexa Fluor 546 antibody (1:200; catalog no. A11075; RRID: AB_2534119; Abcam). The apoptosis rate is expressed by the ratio of insulin/TUNEL/DAPI co-positive cells to total insulin-positive cells (23). For both cell proliferation and apoptosis, the calculation was made using 50 islets from each mouse (4). To visualize mitochondrial density, slides were stained with anti-Tom20 antibody (1:200; catalog no. D8T4N; RRID: AB_2687663; Cell Signaling, Danvers, MA) as well as anti-insulin antibody and DAPI, as mentioned.

#### 2.2.5 Electron microscopy of pancreatic β-cell mitochondria

The resected pancreas was fixed in a solution of 2% glutaraldehyde in 0.1 mM phosphate buffer (pH 7.2) and stored at 4°C for 24 hours. The specimen was postfixed in 2% osmium tetroxide in 0.1 mM phosphate buffer (pH 7.2) for 2 hours at 4°C. Thereafter, the specimen was gradually dehydrated in ethanol and embedded in epoxy resin. The specimen was cut to generate ultrathin sections with an LKB ultramicrotome and mounted on copper grids. The sections were stained with 0.2% lead citrate and 3% uranyl acetate and were observed under TEM (JEM-1400Flash; JEOL, Tokyo, Japan). Analysis of abnormal mitochondria was made on at least 10 β cells of each group. Abnormal mitochondria were defined as swollen mitochondria with disarrayed cristae and a matrix with reduced electron density (24).

### 2.3 *Ex vivo* islet evaluation of imeglimin treatment

#### 2.3.1 Study design

To elucidate the underlying mechanisms of imeglimin’s effect on β cells, 6- and 8-week-old db/db mice were utilized. The 8-week-old db/db mice were treated with 1-week treatment of vehicle, imeglimin, or insulin glargine (Sanofi-Aventis, Paris, France); 6-week-old db/db mice were treated with vehicle for 1 week. In order to clarify the imeglimin’s efficacy on BCM *via* mitochondrial involvement in glucose-dependent or -independent manner, the insulin glargine was subcutaneously injected once daily with dose adjustments to achieve glycemic control comparable to that of the imeglimin-treated group. The mice were sacrificed, and the islets isolated by collagenase digestion, as previously described (25), and subsequently subjected to mitochondrial membrane potential (MMP) and cytosolic cytochrome c measurements. In addition, to assess glucose-dependent and –independent mitochondrial involvement of imeglimin action on β cells, the isolated islets from 6- and 8-week-old db/db mice were incubated in different glucose concentrations of RPMI 1640 (10% FBS, 10 mM HEPES, 5 mM NaHCO_3_, 1 mM sodium pyruvate, 100 mg/ml streptomycin, 100 U/ml penicillin and 11.1 mM or 33.0 mM glucose) with DMSO or 100 µM imeglimin for 24 hours at 37°C and 5% CO_2_ in a humidified incubator, and subjected to MMP measurement with JC-1 dye.

#### 2.3.2 Assessment of mitochondria membrane potential

Measurement of islet MMP was performed according to a previous study (26). Briefly, islets were washed with Krebs-Ringer bicarbonate (KRB) buffer (129.4 mM NaCl, 5.2 mM KCl, 2.7 mM CaCl_2_, 1.3 mM MgSO_4_, 24.8 mM NaHCO_3_, 1.3 mM KH_2_PO_4_, and 2.8 mM glucose), loaded with 1 µL/mL JC-1 dye (catalog no. MT09; Dojindo, Kumamoto, Japan) and incubated at 37°C for 1 hour. The islets were then washed with KRB buffer and evaluated under a fluorescence microscope. In low membrane potential conditions, JC-1 retains its monomer form that emits green fluorescence (500-550 nm); it forms aggregates in high membrane potential conditions that emit red fluorescence (560-510 nm). The fluorescence intensity was analyzed using Fiji software (27). Mitochondria membrane potential is expressed as fold changes of the red to green fluorescence ratio relative to those of 6-week-old control group.

#### 2.3.3 Extraction of the cytosol fraction and measurement of cytochrome c

To assess cytochrome c release, islets were permeabilized to extract the cytosol fraction, as previously described (28). Isolated islets were washed with ice-cold PBS and incubated in 70 µL ice-cold permeabilization buffer [20 mM HEPES, 2.5 mM KH_2_PO_4_, 10 mM KCl, 1.5 mM MgCl_2_, 250 mM sucrose, pH 7.5, with 50 µg/ml digitonin, and protease inhibitors (catalog no. 116974; Roche, Germany)] for 5 min. The islets were then centrifuged at 13,000 x g for 10 min at 4°C. The concentration of cytochrome c in the supernatant and that of total protein were measured using Cytochrome c Quantikine ELISA kit (catalog no. MCTC0; R&D System, Minneapolis, MN) or BCA assay kit for low protein concentration (catalog no. ab207002; Abcam), respectively.

### 2.4 Statistical analysis

Data are expressed as mean ± standard deviation (SD) or mean ± standard error of the mean (SEM), as appropriate. Repeated-measure ANOVA with Fisher’s LSD test was used for longitudinal SPECT/CT and AUC data. Unpaired t-test or Mann-Whitney U-test was employed to compare two groups. Pearson’s correlation was performed for the correlation between pancreatic uptake values and the immunohistological BCM. Statistical analysis and data visualization were carried out with GraphPad Prims 9 software (GraphPad Software, San Diego, CA, USA). Statistical significance was set at *p* < 0.05.

## 3 Results

### 3.1 Imeglimin attenuated progression of glucose intolerance in prediabetic db/db mice

**Figure 2:**
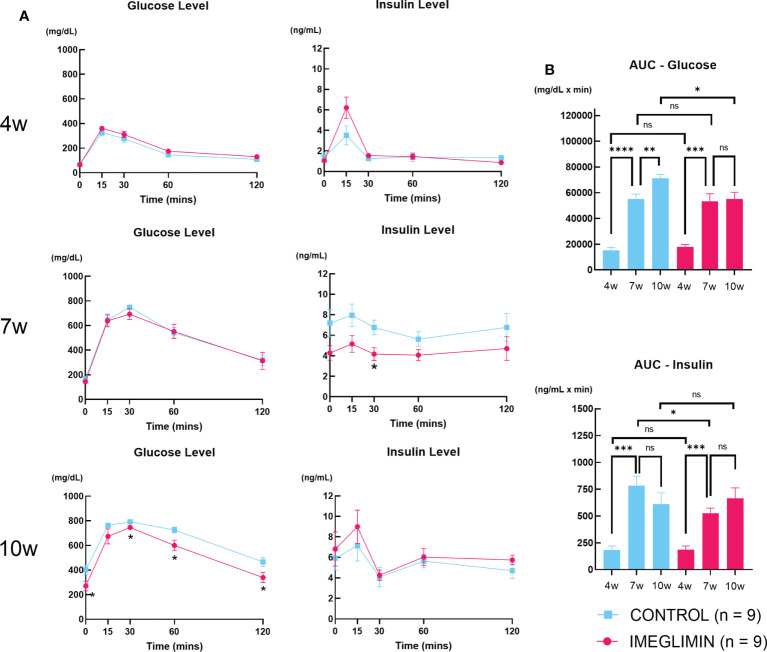
**(A)** Blood glucose and plasma insulin levels and **(B)** AUC of blood glucose and plasma insulin during OGTT at age of 4, 7, and 10 weeks. Control, cyan squares and bars (n = 9); imeglimin-treated, magenta circles and bars (n = 9). Data are presented as mean ± SEM. Control vs imeglimin-treated, *p < 0.05, **p < 0.01, ***p < 0.001, ****p < 0.0001. ns, not significant.

There was no significant difference in non-fasting blood glucose between imeglimin-treated and control groups at baseline. During 5-week intervention, non-fasting blood glucose levels of the imeglimin-treated group increased less than those of the control group. Significant differences emerged after 6 weeks of age (**Figure 1**). Both groups showed body weight gain, but there was no significant difference between imeglimin-treated and control groups during the intervention (**Figure 1**). OGTTs were performed on the imeglimin-treated and control groups at age 4 (baseline), 7, and 10 weeks. There were no statistical differences in blood glucose or plasma insulin levels between the two groups at baseline (**Figure 2**). At 7 weeks of age, the blood glucose levels were not significantly different between the two groups, while the insulin level was significantly higher in control group at 30 mins after glucose administration (*p* < 0.05) (**Figure 2**). At 10 weeks of age, the imeglimin-treated group had significantly lower blood glucose levels at 0, 30, 60, and 120 min after glucose administration compared to those of the control group (*p* < 0.05) (**Figure 2**). The AUC of blood glucose levels of the imeglimin-treated group was significantly lower than that of the control group at 10 weeks of age (*p* < 0.05) (**Figure 2**). The AUC of plasma insulin levels showed a significant difference between the two groups at 7 weeks of age (*p* < 0.05) (**Figure 2**).

### 3.2 Histological analysis indicated preservation of BCM in db/db mice treated with imeglimin

**Figure 3:**
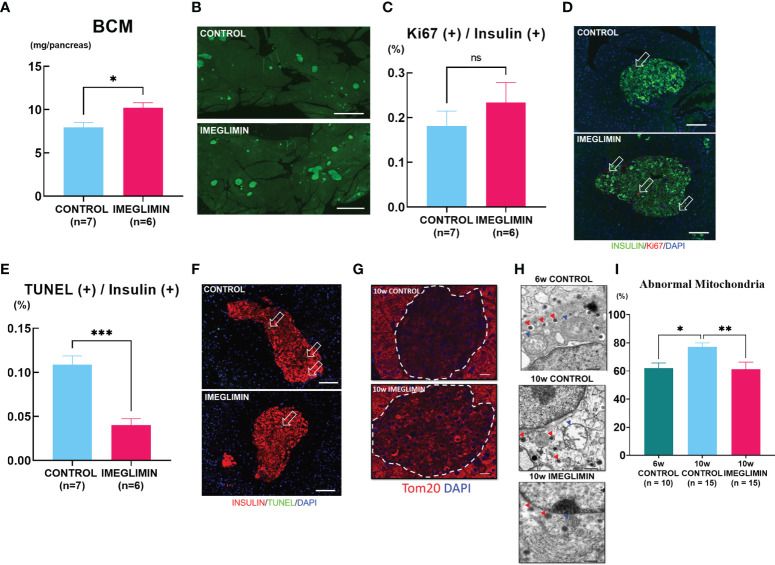
**(A)** BCM of vehicle-treated (n = 7) and imeglimin-treated (n = 6) mice as calculated by immunohistochemistry analysis at age 10 weeks. **(B)** Representative images of insulin-positive area. **(C)** Proliferation rate expressed as the ratio of insulin/Ki67/DAPI co-positive cells to insulin-positive cells in 10-week-old controls (n = 7) and imeglimin-treated mice (n = 6). **(D)** The representative images of insulin/Ki67/DAPI co-positive cells. **(E)** Apoptosis rate expressed as the ratio of insulin/TUNEL/DAPI co-positive cells to insulin-positive cells in 10-week-old controls (n = 7) and imeglimin-treated mice (n = 6). **(F)** The representative images of insulin/TUNEL/DAPI co-positive cells. **(G)** Staining with Tom20 antibody to visualize mitochondrial density in pancreatic β cells. **(H)** Representative images of mitochondria observed by electron microscope (mitochondria, blue triangles; insulin granules, red triangles). **(I)** Ratio of abnormal mitochondria to total mitochondria per β cell of 6-week-old control (10 β cells), 10-week-old control (15 β cells), 10-week-old imeglimin-treated mice (15 β cells). 6-week-old control, teal bar; 10-week-old control, cyan bar; imeglimin-treated, magenta bar. Data are presented as mean ± SEM. Scale bar for **(B)** is 1000 µm, **(D, F)** is 100 µm, **(G)** is 20 µm and **(H)** is 500 nm. *p < 0.05, **p < 0.01, ***p < 0.001. ns, not significant.

The conventional histological analysis of BCM was performed at 10 weeks of age. The imeglimin-treated group showed larger BCM compared to the control group (*p* < 0.05; **Figure 3**). Representative images for each group are shown in **Figure 3**. In the immunohistochemical analysis of the pancreas, no significant difference was observed in the ratio of insulin/Ki67/DAPI co-positive cells to total insulin-positive cells between the imeglimin-treated mice and controls (**Figure 3**). On the other hand, the imeglimin-treated group had a drastically reduced ratio of insulin/TUNEL/DAPI co-positive cells to total insulin-positive cells compared with that of the control group (*p* < 0.001; **Figure 3**). To clarify the influence of imeglimin on the mitochondria of pancreatic β cells, mitochondrial density in the islets was visualized by immunostaining with anti-Tom20 antibody. As seen in **Figure 3**, the imeglimin-treated group showed a relatively higher Tom20 density compared to that of the control group. Electron microscope analysis of the pancreatic β cells obtained from 6- and 10-week-old db/db mice with and without *in vivo* imeglimin treatment was also performed. Representative electron microscopy images of mitochondria in β cells are shown in **Figure 3**. We found that the 10-week-old control group had a significantly higher proportion of abnormal swollen mitochondria exhibiting loss of structured cristae compared to that of both the 6-week-old control group and the 10-week-old imeglimin-treated group (*p* < 0.05 and *p* < 0.01, respectively) (**Figure 3**).

### 3.3 Imeglimin preserved BCM as assessed by non-invasive longitudinal BCM analysis using ^111^In-exendin-4 SPECT/CT

**Figure 4:**
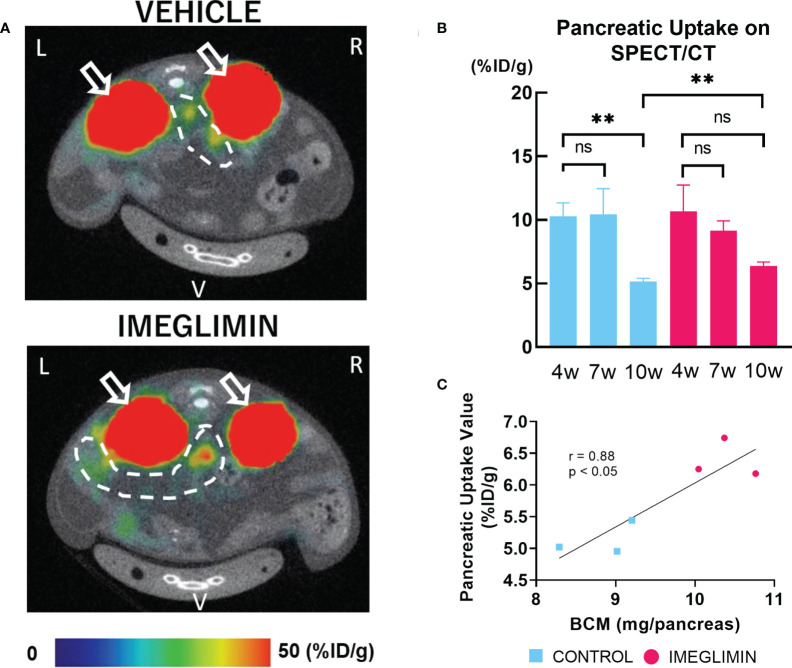
**(A)** Representative axial images of ^111^In-exendin-4 SPECT/CT of 10-week-old db/db mice. Signals from the kidney, white arrow; signal from pancreas, dashed white circle. L, left; R, right; V, ventral. **(B)** Pancreatic uptake values (%ID/g) detected by multiple SPECT/CT scans at age 4, 7, and 10 weeks. Control, cyan bars (n = 3); imeglimin-treated, magenta bars (n = 3). **(C)** Correlation between pancreatic uptake value calculated by ^111^In-exendin-4 SPECT/CT and BCM measured by immunohistochemistry in 10-week-old mice (n = 6). Data are presented as mean ± SD. **p < 0.01. ns, not significant.

The ^111^In-exendin-4 SPECT/CT technique was performed on imeglimin-treated and control groups at age 4, 7, and 10 weeks. Representative images of SPECT/CT for both groups at 10 weeks of age are shown in **Figure 4**. The baseline pancreatic uptake values at 4 weeks of age were not significantly different between the two groups (**Figure 4**). The control group showed a significant decrease in pancreatic uptake value at the end of the study period (10 weeks of age) (*p* < 0.01). A more gradual decrease in pancreatic uptake value was observed in the imeglimin-treated group, although there was not a significant difference (**Figure 4**). Furthermore, by the age of 10 weeks, the pancreatic uptake values were significantly greater in the imeglimin-treated group than those in the control group (*p* < 0.01) (**Figure 4**), which was consistent with histological analysis (**Figure 3**). In addition, we found a statistically significant correlation between pancreatic uptake values obtained from SPECT/CT scans and histologically calculated BCM at 10 weeks of age (r = 0.88; *p* < 0.05; **Figure 4**).

### 3.4 *In vivo* treatment with imeglimin improved mitochondrial function of db/db islets

**Figure 5:**
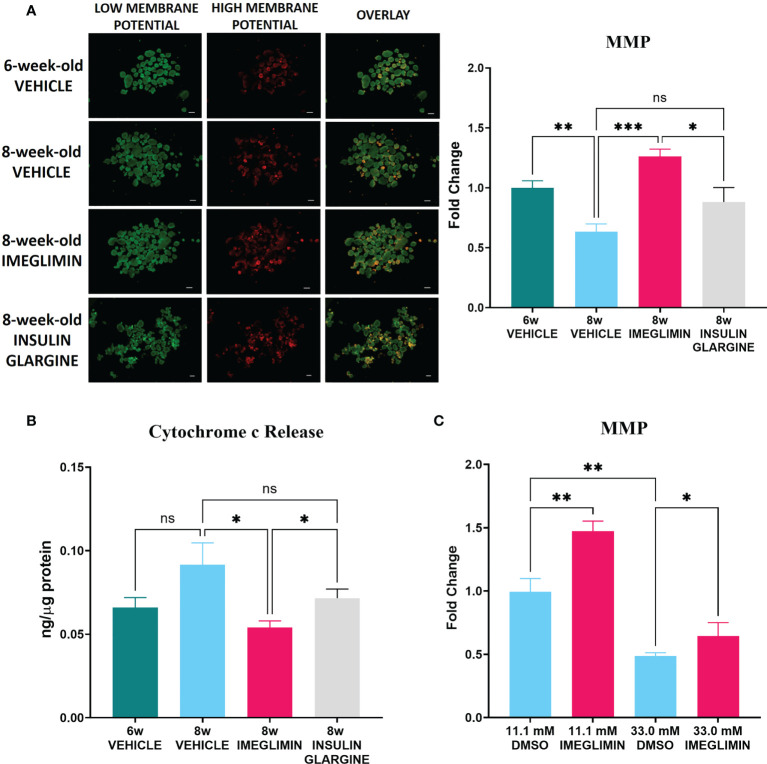
**(A)** Representative images (left) and intensity ratio of red/green fluorescence (right) of JC-1-stained islets for measurement of MMP (n = 5). Data are presented as mean ± SEM. **(B)** The concentration of released cytosolic cytochrome c normalized with total protein in islets (n = 5-6). Mice were treated with vehicle or imeglimin for 1 week before islet isolation. **(C)** The ratio of red/green fluorescence of islets isolated from 6-week-old mice were subsequently incubated in RPMI containing 11.1 mM or 33.0 mM glucose with DMSO or 100 µM imeglimin for 24 hours (n = 6). Data are presented as mean ± SEM. Scale bar, 200 µm. *p < 0.05, **p < 0.01, ***p < 0.001. ns, not significant.

To further evaluate mitochondrial involvement in the effects of imeglimin on β-cell preservation, isolated islets obtained from 1-week vehicle-, imeglimin-, or insulin glargine-treated db/db mice at 6 and 8 weeks of age were subjected to measurement of MMP and released cytosolic cytochrome c protein levels. The age selections were decided based on (1) non-fasting blood glucose, which began showing a significant difference after 6 weeks of age (**Figure 1**) and (2) the drastic BCM loss detected by ^111^In-exendin-4 SPECT/CT, which first appeared at 8 weeks of age (**Figure 4**). In the analysis of MMP, images of islets dyed with JC-1 are shown in **Figure 5**. The images reveal that islets isolated from the 6-week-old control group had higher MMP compared to that of the 8-week-old vehicle-treated group (*p* < 0.01) and also that the 8-week-old imeglimin-treated group had higher MMP compared to the 8-week-old vehicle-treated and insulin glargine-treated groups (*p* < 0.001 and *p* = 0.03, respectively) (**Figure 5**). The cytosolic cytochrome c protein level was then measured by ELISA on the cytosolic fraction of isolated islets. The 6-week-old vehicle-treated group tended to show a lower released cytosolic cytochrome c level compared with that of the 8-week-old vehicle-treated group, while the 8-week-old imeglimin-treated group showed significantly lower released cytochrome c level than the 8-week-old vehicle- and insulin glargine-treated groups (*p* = 0.03 and *p* = 0.03, respectively) (**Figure 5**). These findings are consistent with the immunohistochemical results of TUNEL analysis in long-term *in vivo* treatment with imeglimin in db/db mice (**Figure 4**). To analyze the glycemic influence on β-cell preservation by imeglimin, islets from mice aged 6 weeks were isolated and subsequently incubated in RPMI 11.1 mM glucose with DMSO or 100 µM imeglimin, and 33.0 mM glucose with DMSO or 100 µM imeglimin for 24 hours. Islets incubated in 11.1 mM glucose with imeglimin demonstrated significantly higher MMP compared to islets incubated in 11.1 mM glucose with DMSO (*p* < 0.01). Although islets in 33.0 mM glucose with DMSO showed significantly lower MMP than those in 11.1 mM glucose with DMSO (*p* < 0.01), islets incubated in 33.0 mM glucose with 100 µM imeglimin exhibited significantly higher MMP compared to 33.0 mM glucose with DMSO (*p* = 0.01) (**Figure 5**).

## 4 Discussion

In the present study, we investigated the effects of imeglimin on BCM of prediabetic db/db male mice. During the 5-week study period, treatment with imeglimin resulted in greater preservation of BCM and attenuated the progression of glucose intolerance in comparison with mice treated with vehicle. Preservation of BCM was ascertained noninvasively and longitudinally by use of the^111^In-exendin-4 SPECT/CT imaging technique. Moreover, in the immunohistological analysis, 5-week treatment with imeglimin was found to reduce β-cell apoptosis in db/db mice, which is consistent with the lower levels of cytochrome c release, a marker of mitochondria-mediated apoptosis, seen in islets isolated from mice after 1-week *in vivo* imeglimin treatment. Electron microscopy revealed preserved structures of mitochondrial cristae and outer membrane in the imeglimin-treated mice, together with consequentially lower ratios of abnormal mitochondria in pancreatic β cells compared to those in the control group. *In vivo* imeglimin administration also demonstrated preservation of MMP in islets of db/db mice, while *ex vivo* imeglimin administration showed the preservation to be partly independent of the glucose level. These findings suggest that the preservative effect of imeglimin on BCM is mediated *via* β-cell mitochondrial involvement.

During the 5-week period of *in vivo* intervention, we detected longitudinal changes of BCM in db/db mice using ^111^In-exendin-4 SPECT/CT imaging. The ^111^In-exendin-4 SPECT/CT technique obviated several limitations of immunohistochemical BCM measurement, such as the limitation to cross sectional data (5, 6), the lack of staining uniformity (6) and the use of a restricted sampling area to represent whole pancreas BCM (6). Several radioisotope-labeled exendin-based probes have been developed for noninvasive β-cell imaging and evaluation of BCM in living subjects (6, 29), including our recently developed ^111^Indium-labeled [Lys12(111In-BnDTPA-Ahx)]exendin-4) (^111^In-exendin-4) technique (5). In earlier studies, ^111^In-exendin-4 SPECT/CT was applied to NOD and db/db mice with remarkable consistency with histologically determined BCM (4, 9, 10). Based on the results of our ^111^In-exendin-4 SPECT/CT analysis, the imeglimin-treated group showed larger BCM at 10 weeks of age compared to that of the control group (**Figure 4**), demonstrating the preservative effect on BCM of *in vivo* treatment with imeglimin. Furthermore, our findings by ^111^In-exendin-4 SPECT/CT analysis also display a strong correlation with BCM measured by conventional immunohistochemistry at the age of 10 weeks (**Figure 4**). These results are consistent with a previous report showing that oral administration of imeglimin attenuated BCM loss and improved glycemic control in ZDF rats, an obesity-driven T2DM model (30). Interestingly, in imeglimin-treated mice, no prominent BCM change was detected by ^111^In-exendin-4 SPECT/CT between the ages of 4 and 10 weeks, indicating attenuated BCM loss compared with control. On the other hand, in control mice, ^111^In-exendin-4 SPECT/CT revealed that BCM loss occurred mainly between 7 and 10 weeks of age. This concurs with another conventional immunohistochemical study on db/db mice showing that most of the loss of BCM occurs between 6 and 10 weeks of age (21). In this study, insulin secretion was increased at 7 weeks but it tended to be reduced at 10 weeks in control mice, although the AUC_glucose levels increased significantly at 10 weeks compared with 7 weeks of the age (**Figure 2**). These results were consistent with the natural course of diabetes development in db/db mice (17). On the other hand, in imeglimin-treated group, increasing tendency of insulin secretion was found throughout 6-week observation while the AUC_glucose levels didn’t show significant differences between 7 and 10 weeks of the age (**Figure 2**). The findings suggested the attenuation of diabetes development and progression in imeglimin-treated mice compared with control mice. Thus, our study demonstrates the utility of ^111^In-exendin-4 SPECT/CT in clearly identifying distinct patterns of longitudinal changes in BCM in prediabetic db/db mice with and without imeglimin treatment.

Our immunohistochemistry analysis reveals preservation of BCM by imeglimin is driven mainly by inhibition of apoptosis of β cells (**Figures 3A**). It has been reported that db/db mice develop progressive loss of BCM due to increased frequency of apoptosis of β cells as well as their reduced proliferation (31). We found a tendency to a higher ratio of insulin/Ki67/DAPI co-positive cells after 5-week intervention with imeglimin. In addition, significantly decreased frequency of apoptotic β cells were detected in imeglimin-treated mice. A previous study showed that imeglimin hinders a rising apoptosis rate by inhibiting mitochondrial permeability transition pore opening and cytochrome c release in human endothelial cells (32). Similarly, we found a lower cytosolic concentration of released cytochrome c in islets isolated from db/db mice after 1-week *in vivo* treatment by imeglimin (**Figure 5**). In the mitochondrial-mediated apoptosis cascade, the release of cytochrome c into cytosol triggers the formation of the apoptosome and activates pro-apoptotic enzymes (33). Thus, our findings suggest that imeglimin inhibits mitochondria-mediated apoptosis in β cells under 5-week *in vivo* treatment, which then may contribute to preservation BCM of db/db mice.

In-depth analyses of the role of mitochondria in imeglimin’s action on β cells were performed by evaluating mitochondrial structure and MMP. The db/db mice have been characterized with excessive mitophagy in pancreatic β cells, which is a mechanism for breaking down defective mitochondria (21). By utilizing TEM, our examination of β-cell mitochondria revealed lower frequencies of mitochondrial structure abnormality in 5-week imeglimin-treated db/db mice compared to that of control. The structural integrity of mitochondria was also associated with mitochondrial activity; 1-week imeglimin treatment in 8-week-old db/db mice improved islet MMP to a level comparable to islet MMP from 6-week-old mice, which is significantly higher than that of vehicle and insulin glargine (**Figure 5**). Several studies have reported that imeglimin improved mitochondrial condition by increasing the NAD^+^ concentration (18), reducing radical oxygen species, and increasing production of cardiolipin (34). Cardiolipin has been reported to not only organize membrane structures such as cristae and contact sites, but also plays a role in respiration and energy conversion (35). Moreover, to identify glucose dependency and independency of imeglimin action in inhibiting mitochondrial-mediated β-cell apoptosis, isolated islets obtained from 6-week-old db/db mice were incubated in 11.1 mM or 33.0 mM glucose with DMSO or imeglimin (**Figure 5**). We demonstrated a significant decrease of MMP in islets incubated in 33.0 mM glucose compared to those incubated in 11.1 mM glucose. Nevertheless, the addition of imeglimin to 33.0 mM glucose partly restored MMP in islets. These findings imply that imeglimin’s effect on islet mitochondria is, at least partially, independent of the glucose level. Previous studies reported that imeglimin exhibited a similar glucose-independent effect on survival of rat INS-1 cells (16) and islets isolated from wild-type C57BL/6J mice (19). Therefore, it is suggested that imeglimin inhibits apoptosis by improving the structure of mitochondria and the functional conditions in both a glucose-dependent and –independent manner in db/db mice.

Finally, the number of mice for the SPECT/CT analysis in the present study was relatively small. This might inhibit a more robust conclusion on the effectiveness of ^111^In-exendin-4 SPECT/CT in observing BCM changes over time. The application of ^111^In-exendin-4 SPECT/CT with imeglimin treatment to different mice strains as well as human subjects need to be explored. Additionally, since this study was carried out in pre-diabetic mice, careful interpretation should be noted for the exact influence on BCM by imeglimin in diabetic subjects. Although we primarily focused here on imeglimin’s efficacy in preserving BCM and the mechanisms of its action from the β-cell perspective, the favorable effects on gluconeogenesis and insulin sensitivity have been reported (19). Interestingly, in this study, reduced insulin secretion at 7 weeks of age was observed in imeglimin-treated group compared with control group (**Figure 2**). Considering no significant differences of AUC_glucose levels at 7 weeks of age between the two groups (**Figure 2**), imeglimin treatment might contribute to the improvement of insulin sensitivity and/or the attenuation of the increasing insulin resistance. Further study to comprehensively understand systemic imeglimin’s efficacy in type 2 diabetes mellitus is required.

In summary, we demonstrated that longitudinal observation using ^111^In-exendin-4 SPECT/CT, in combination with metabolic observations, provides comprehensive evidence on imeglimin action to attenuate the course of diabetes development in db/db mice. These findings open possibilities of longitudinal BCM measurement not only in evaluation of novel anti-diabetes treatments, but also in obtaining insight of diabetes pathogenesis in different animal models. In addition, we discovered that imeglimin prevents β-cell apoptosis by improving the mitochondrial structure and membrane potentials of β cells. Thus, imeglimin may well have beneficial effects on β-cell mitochondria *via* improvement of glycemic control as well as glycemic-independent fashions.

## Data availability statement

The raw data supporting the conclusions of this article will be made available by the authors, without undue reservation.

## Ethics statement

The animal study was reviewed and approved by The Committee of Animal Care and Use, Kyoto University (approval No. MedKyo 21508, 22222, 22227).

## Author contributions

MF and TM planned the study, acquired data, analysed data, wrote, reviewed and edited the manuscript. HF and MO contributed to discussion. AB, KS, and SK participated in data acquisition. NI contributed to discussion, reviewed the manuscript, and supervised the project. All authors approved the final version of the manuscript.

## Funding

This study was supported by grants from the Ministry of Education, Culture, Sports, Science and Technology (MEXT), Japan Society for the Promotion of Science (JSPS) (grant numbers 21K20931, 21K08553, 22K16411), Japan Association for Diabetes Education and Care, Japan Diabetes Foundation, the Japan Foundation for Applied Enzymology (Front Runner of Future Diabetes Research), and MSD Life Science Foundation.

## Acknowledgments

The authors thank Dr. Tatsuya Katsuno (Center of Anatomical, Pathological and Forensics Medical Researches, Kyoto University) for assistance on electron microscopy. The authors also thank Takashi Nishimoto and Norinaga Kakishita (Radioisotope Research Center, Kyoto University) for assistance on radioisotope study and SPECT/CT scan.

## Conflict of interest

NI received joint research grants from Daiichi Sankyo Co., Ltd., Terumo Co., Ltd., and Drawbridge Health, Inc.; received speaker honoraria from Kowa Pharmaceutical Co., Ltd., MSD, Astellas Pharma Inc., Novo Nordisk Pharma Ltd., Ono Pharmaceutical Co., Ltd., Nippon Boehringer Ingelheim Co., Ltd., Takeda Pharmaceutical Co., Ltd., and Mitsubishi Tanabe Pharma Co., Ltd.; received scholarship grants from Kissei Pharmaceutical Co., Ltd., Sanofi, Daiichi-Sankyo Co., Ltd., Mitsubishi Tanabe Pharma Co., Ltd., Takeda Pharmaceutical Co., Ltd., Japan Tobacco Inc., Kyowa Kirin Co., Sumitomo Pharma Co., Ltd., Astellas Pharma Inc., MSD, Eli Lilly Japan, Ono Pharmaceutical Co., Ltd., Sanwa Kagaku Kenkyusho Co. Ltd., Nippon Boehringer Ingelheim Co., Ltd., Novo Nordisk Pharma Ltd., Novartis Pharma K.K., Teijin Pharma Ltd., and Life Scan Japan Inc.

The remaining authors declare that the research was conducted in the absence of any commercial or financial relationships that could be construed as a potential conflict of interest.

## Publisher’s note

All claims expressed in this article are solely those of the authors and do not necessarily represent those of their affiliated organizations, or those of the publisher, the editors and the reviewers. Any product that may be evaluated in this article, or claim that may be made by its manufacturer, is not guaranteed or endorsed by the publisher.
